# Rechargeable lithium-ion cell state of charge and defect detection by in-situ inside-out magnetic resonance imaging

**DOI:** 10.1038/s41467-018-04192-x

**Published:** 2018-05-03

**Authors:** Andrew J. Ilott, Mohaddese Mohammadi, Christopher M. Schauerman, Matthew J. Ganter, Alexej Jerschow

**Affiliations:** 1 Department of Chemistry, New York University, 100 Washington Square East, New York, NY 10003 USA; 2 The Battery Prototyping Center, Rochester Institute of Technology, 156 Lomb Memorial Drive, Rochester, NY 14623 USA

## Abstract

When and why does a rechargeable battery lose capacity or go bad? This is a question that is surprisingly difficult to answer; yet, it lies at the heart of progress in the fields of consumer electronics, electric vehicles, and electrical storage. The difficulty is related to the limited amount of information one can obtain from a cell without taking it apart and analyzing it destructively. Here, we demonstrate that the measurement of tiny induced magnetic field changes within a cell can be used to assess the level of lithium incorporation into the electrode materials, and diagnose certain cell flaws that could arise from assembly. The measurements are fast, can be performed on finished and unfinished cells, and most importantly, can be done nondestructively with cells that are compatible with commercial design requirements with conductive enclosures.

## Abstract (web summary)

The development of noninvasive methodology plays an important role in advancing lithium ion battery technology. Here the authors utilize the measurement of tiny magnetic field changes within a cell to assess the lithiation state of the active material, and detect defects.

## Introduction

Batteries are a crucial enabling technology in many important energy solutions and they are integral to advances in portable electronics, electric vehicles, and grid storage. Continued demand for batteries with high-energy capacity and the desire to quickly charge and discharge the devices present a number of formidable engineering and scientific challenges. Ensuring device safety is an important consideration, which needs to be addressed with care. Several industry leaders have experienced unforeseen setbacks due to battery and cell malfunctions, such as most recently, for example, seen in the Samsung Note 7 devices or in the iPhone 8 swelling issues. One major reason for the recurrence of such problems, and for the slow progress in battery technology is the difficulty in tracking defects inside the cells during operation in a nondestructive fashion.

X-ray CT^1,2^ is a successful technique for scanning electrochemical cells, but it is relatively slow, and thus usually not applicable for high throughput or in situ applications. Furthermore, X-ray CT provides diagnostics mostly of the denser components of a cell, and does not offer insights into subtle chemical or physical changes of the materials inside. A recently developed acoustic technique^3^ appears to be a highly promising methodology for the nondestructive characterization of cell behavior throughout the cell life, and is currently being investigated for its sensitivity to important cell behavior.

Magnetic resonance (MR) techniques have been developed to measure several different cell properties^4–13^. A fundamental limitation that is difficult to overcome under typical operating conditions is that conductors are not transparent to rf irradiation. Often, the cell casing is made of conductive material, such as polymer-lined aluminum in pouch or laminate cells, but also the electrodes preclude the use of conventional MR for realistic or commercial-type cell geometries. Nonetheless, MR has provided important insights into electrolyte behavior, Li-dendrite growth, and other electrochemical effects by the use of custom-built cells, which allow convenient rf access^4–13^.

Here, we demonstrate an MR technique, which overcomes these limitations, and provides cell diagnostics without requiring rf access to the inside of the cell. The technique is based on imaging the induced or permanent magnetic field produced by the cell, and connecting it with processes occurring inside the cell. The reason that this magnetic field is so informative, is that the magnetic susceptibility χ is material dependent, and that the resulting magnetic field is dependent on the distribution of the materials inside the cell, which can change during cell operation.

The magnetic susceptibility also depends on the electronic configuration of the material, and hence during redox reactions, such as battery charging or discharging, there can be large changes in magnetic susceptibility. Measurements of magnetic susceptibility can therefore yield detailed information about the oxidation state of the materials inside an electrochemical device to give insights into the state of charge^14^ (SOC) of the battery and its failure mechanisms. Furthermore, the magnetic susceptibilities of many widely used electrode materials, including, for example, Li_*x*_MnO_2_^15^, Li_*x*_FePO_4_^14^, Li_*x*_CoO_2_^16^, and Li_*x*_Ni_*y*_Mn_*y*_Co_1−2*y*_O_2_^17^, depend upon their lithiation state. Graphite, a popular anode material, is strongly diamagnetic and has a highly anisotropic susceptibility. In this case, as Li^+^ intercalates into the structure, the interlayer distance in graphite increases and the susceptibility and its anisotropy are significantly reduced. This effect is highly dependent on the stage (the number of graphite layers between each lithium layer) of the resulting lithium intercalate^18^.

Monitoring the magnetic field produced by the cell when it is placed into an external magnetic field thus offers the ability to monitor electrochemical processes in situ. Moreover, the distribution of magnetic material inside the cell influences the spatial variation in the magnetic field that it produces, such that it is also sensitive to the precise construction of the cell. In this manner, measures of the magnetic field can be used to screen for physical defects in cells.

MR methods provide the ability to measure tiny changes in magnetic field maps^19^, for example, through the use of phase-map imaging or specific NMR probes^20^. In the phase-map imaging approach, multiple images are acquired at different echo times (TEs) and used to reconstruct the spatial variation in the induced resonance frequency shift from the evolution of the signal phases. In this manner, very accurate field maps can be obtained—sensitivity of the order of μT. Since ultimately, the magnetic field changes are measured, apart from measuring the magnetic properties of a device, one could also measure current distributions in the same manner, which could arise, for example, in the relaxation phase between charging steps, or during charging or discharging itself.

In this paper, we show how the magnetic field can be accurately measured by MR around a Li-ion pouch cell. As the cell is charged, the field changes in a highly predictable manner. The results can be used, for example, to infer the average oxidation state of the cathode component as a function of charge. In order to demonstrate the potential of this technique, it is important to know the precise construction of a cell and the materials used, and to explore different types of defects that could happen during construction. Therefore, we have measured cells which were in pristine condition but also those that had specific flaws introduced into them. This approach allowed us to demonstrate the identification of certain types of these defects by the noninvasive MR method.

## Results

### Diagnosing state of charge

**Fig. 1:**
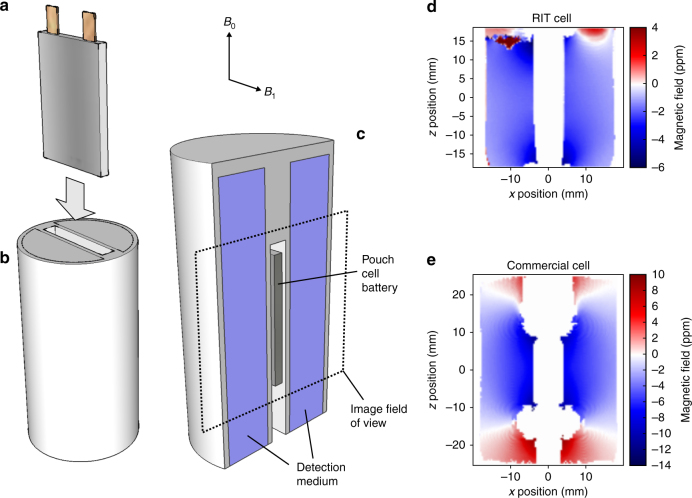
Magnetic field map measurements for the fully charged cells. **a**–**c** Sample placement, and image orientation; **d**,**e** field maps measured for the cells. Field maps are referenced here to the empty holder, giving an absolute field map for the Li-ion cell

Figure 1 shows the schematic of the setup for pouch-cell imaging and the associated magnetic field map obtained from a fully charged Li-ion pouch cell. Only the magnetic field in the plane perpendicular to the main face of the cell is displayed for clarity. The map shows a 1–2-ppm change in the field due to the magnetic properties of the battery. This change in field is large in comparison to the typical resolution limit of phase-mapping methods, where it has been demonstrated that differences in susceptibilities of 0.1 ppm (or about 1 μT) can be resolved easily^21^. The method is insensitive to changes in the background field or fluctuations in the instrument’s magnetic field because all measurements can be taken with respect to a reference image of the holder alone, a reference cell, or the initial state of the same cell.

There are artifacts at the corners of the field map, as expected, where the magnetic properties change particularly rapidly, which is also due to the presence of the leads and air pockets, but these effects are short ranged and these regions can be neglected.

**Fig. 2:**
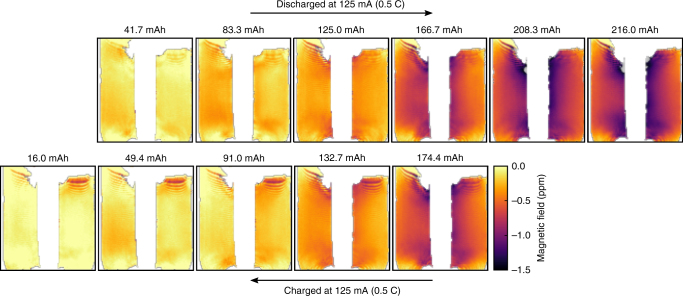
Series of magnetic field maps taken at intervals during discharge and then charge of the cell. The plots are labeled by the discharge capacity of the cell at each step. The magnetic field maps are referenced to the field map produced by the fully charged cell. The RIT cell was used for this purpose, and the susceptibility increased upon discharge

We now demonstrate that the magnetic field can be used as a diagnostic for a cell’s SOC and to measure defects in a cell’s construction. Fig. 2 shows the change in the field map measured around the cell at discrete steps during discharge and then charge. The maps show that the field gradually reduces during discharge, to a minimum of –1.5 ppm (14.1 μT) lower than in the fully charged cell, with the reverse trend followed on the subsequent charge steps. The changes occur in a mostly symmetric fashion, with two symmetry planes bisecting the map vertically and horizontally.

Each step in the charge/discharge profile in Fig. 2 results in a unique field map, with changes that can be readily measured. This one-to-one mapping between charge state and the measured field map can therefore provide a fast tool for recovering the SOC of an unknown cell, which may not be available from voltage measurements for many cell types, especially if the cell is compromised. More importantly, because it is the variation in the oxidation states of the anode and cathode materials that drive the differences in the measured bulk magnetic susceptibility, the field maps can provide vital additional information about cell health by providing a measure of the parameter distributions.

**Fig. 3:**
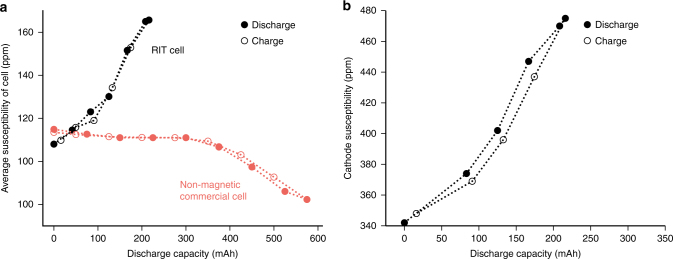
Fitted magnetic susceptibilities of the cell during the discharge/charge. For simplicity, the cell is assumed to have a uniform volume susceptibility in **a**. Error bars are smaller than the size of the points. Knowing the cell construction, one can, however, also determine the average susceptibility of the cathode material in **b**. Details of the calculations are shown in Supplementary Table 1, Supplementary Table 2, and the Methods section. The susceptibility values are given in ppm indicating a factor of 10^–6^

From these data, one can derive the cell’s susceptibility changes over the charge cycle. Fig. 3 shows how the bulk magnetic susceptibility changes during discharge for two types of cells. The RIT cell uses NMC (Li_*x*_Ni_*y*_Mn_*y*_Co_1−2*y*_O_2_) as the cathode material, and it is known that the magnetic susceptibility of this material increases with lithiation level^17^. By contrast, results are also shown for a commercial cell with LCO (Li_*x*_CoO_2_) as the cathode material. In that case, susceptibility decreases upon cathode lithiation (discharge), as described in the literature^16^. Since the trends go in opposite directions, the selection of these materials formed an important test of the methodology. Both of these effects are clearly observed over a full cycle in Fig. 3a, and can be measured precisely. The analysis shown in Fig. 3a is based on the simplest-possible model, the susceptibility being distributed uniformly across the cell. Knowing the geometry and the materials of the cell, however, one can obtain a more detailed analysis and extract the susceptibility changes of the cathode materials alone, the results of which are shown in Fig. 3b. In this analysis, it was determined and observed that the bulk of the susceptibility changes were due to the cathode material, which is often the case, since the other battery components typically only show small variations. Details of the calculation are provided in the Methods section.

### Detecting cell defects

**Fig. 4:**
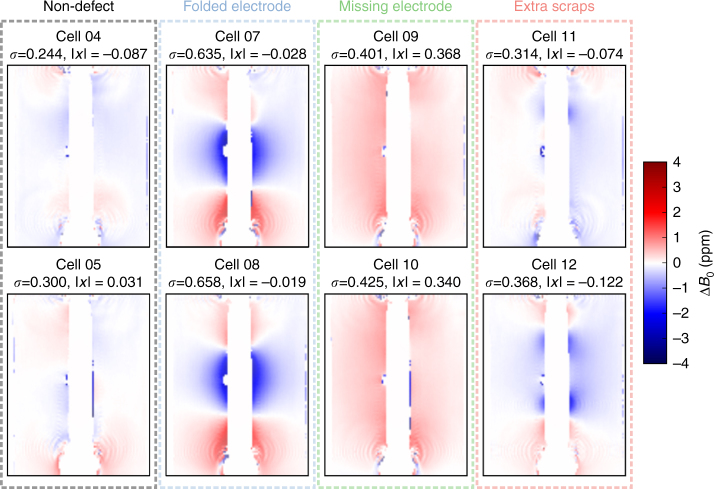
Magnetic field maps for the defective cells, with the mean and standard deviation indicated (taken over all the voxels in each image). The fields are given relative to one of the non-defect cells

Both the spatial dependence of the oxidation state, and the distribution of the material in space also affect the bulk magnetic susceptibility. Therefore, this methodology can be used to detect changes in the cell over time, as well as physical defects in a cell. Figure 4 shows the resulting field maps when the method is applied to a series of pouch cells that are purposely defected by folding one of the cathodes, removing a cathode altogether, or adding small scraps of electrode material into the cell construction. Not only do the measured field maps show strong differences that are indicative of the defect types, the observed changes are also intuitive and diagnostic. For example, when the electrode is folded, new features are observed in the image at the locations where the fold occurs. When there is a missing cathode, the mean value of the field noticeably increases, as would be expected. The changes are subtler when extra scraps are added to the cell, but there is a slight increase in the mean and standard deviation of the measured magnetic field. Furthermore, the MRI method is sensitive enough to resolve significant differences for even the two cells prepared without defects. These small differences may not result in critical cell failures but could still affect overall cell capacity and performance, and detecting such differences could help in diagnosing reproducibility in cell construction. This additional information could be leveraged by correlating data from a large number of cell magnetic field maps with their synthesis/manufacturing conditions and electrochemical performance. It should be noted that the susceptibility measurements shown here can also be performed with cells that are not fully finished (i.e., which do not contain electrolytes and did not undergo a formation cycle), and thus, a manufacturer could potentially avoid a costly finishing and formation cycle of cells that are shown to be defective at an early stage. The defective cells studied here were unfinished (without electrolyte) to illustrate this point.

**Fig. 5:**
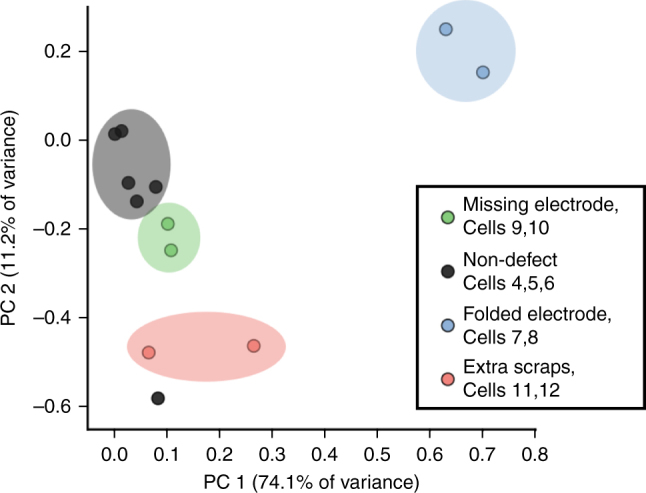
Principal component analysis performed on the magnetic field maps of the cells shown in Fig. 4. This analysis shows the grouping of cells into normal cells and cells with particular defects

Although many defects are clearly visible and interpretable directly from the field maps (Fig. 4), further opportunities arise when one considers a potentially high-throughput application. The measurements are sufficiently fast to be performed on a large number of cells, and the results could be correlated with additional cell characteristics to predict cell differences based on subtle features in the maps. To illustrate this point, we have performed a principal component analysis (PCA) using the 2D images from Fig. 4 on the limited number of samples available here. The PCA score plot is shown in Fig. 5, where a strong grouping can be seen for each of the cell types. Interestingly, while it could be difficult to visually differentiate between the non-defected cells and those with extra scraps, the PCA shows a clear grouping and separation using the second principal component.

In this example, the PCA was performed on the 2D magnetic field maps which are themselves reconstructed from multiple phase-map images. In this kind of analysis, there is no requirement for the input data to be a coherent image. Instead, optimized experiments could be designed that sample the regions of k-space that are expected to vary most strongly. In this manner, the diagnostic power of the experiments could be preserved (or even improved) while drastically reducing the overall experiment time. This latter approach could further benefit from a big data approach when analyzing large numbers of cells, in which machine-learning algorithms could be used to more efficiently classify cells by defect type. In this way, one could further enhance the information content of the observed magnetic field maps.

## Discussion

We have demonstrated here the opportunities of MR-based diagnostics for assessing the state and quality of rechargeable Li-ion cells. The technique is fast, nondestructive, and is based on measuring the small induced and permanent magnetic field changes around a cell. RF penetration into the cell is not required, and thus commercial-style conductive cell enclosures are no impediment to this analysis. We have shown that any overall susceptibility changes can be observed, and that the variation in the cathode susceptibility, related to the lithiation state of the active material, in particular, appears to give rise to the largest change in the charging cycle in the cells studied. Furthermore, we have explored the possibility of detecting defects in cells, which could be determined even in unfinished cells. Particularly interesting future applications could include studies of capacity fade, cell examination after many cycles, and high stress and accelerated aging testing. Certain limitations of the technique should be mentioned here: while in principle there is no limitation on the cell size that can be studied, this does depend on the MR probe and magnet bore sizes. Temperature ranges likewise will depend on the MR hardware used, but it is straightforward to extend the range from –20 to 100 °C, for example. In our study, we determined that NMC and LCO represented the largest contributions to the susceptibility changes, and hence the partial SOC of the cathode was accessible here. Other constant contributions to the magnetic susceptibility can be subtracted out via a reference measurement, but if significant changes of high-susceptibility components were to occur, the isolation of the cathode state may become more difficult. In such cases, it would still be possible to measure the overall changes in susceptibility, but the interpretation of the results would be different. The measurement time is currently 2.5 min, but could easily be reduced to several seconds, in particular if statistical analysis of incompletely sampled data were used. Such implementations would also make this technique compatible with a high volume, automated, and fast screening implementation. Overall, it is hoped that this noninvasive methodology will provide much-needed tools for the development of new battery materials and cell designs that address current and future needs.

## Methods

### Commercial cell

The commercial cell used was a non-magnetic lithium polymer cell, PGEB-NM053040, which was purchased from Powerstream Technology group. It has a capacity of 600 mAh and 3.7 V nominal voltage.

### RIT cells

Multilayer plain-stack cells were manufactured at the RIT battery prototyping center. They were composed of five double-sided cathodes (with copper current collectors) and six double-sided anodes (with aluminum current collector). The cells contained ca. 0.95 mL of 1.2 M LiPF_6_ EC:EMC 3:7 electrolyte. See Supplementary Fig. 1 and Supplementary Table 1 for more detailed information about the composition and dimensions of the cell components.

### Battery holder

The holder was designed in Sketchup and 3D printed in PLA filament in the shape of a cylinder with 39-mm diameter and 77-mm length. See Fig. 1 for a schematic of the holder design. The void space was filled with water as the detection medium.

### Charging conditions and equipment

RIT cell: The cells were initially fully charged to 4.2 V using a constant current of 25 mA (0.1 C). The cells were then discharged in discrete steps at 125 mA (0.5 C) for 20 min until the cell voltage reached 2.5 V. The cell was then charged at 125 mA (0.5 C) in 20-min steps until the cell voltage reached 4.2 V. The MRI experiments were performed after each charge/discharge step after allowing the cell to rest for 15 min. A Biologic VSP potentiostat was used for the electrochemical cycling. The cells were charged and discharged inside the MRI machine without removing them.

### MRI experiments

The MRI experiments were performed on a Bruker Ultrashield 9.4T Avance I spectrometer containing a Bruker Mini0.75 gradient assembly and operating at 400.1 MHz for ^1^H. A Bruker MiniWB57 imaging probe was used to collect all of the data, with a Bruker WB57 40-mm i. d. coil insert for ^1^H experiments. 2D ^1^H MRI experiments were performed using a slice-selective 2D FLASH sequence implemented in Paravision 5.1, using a hermite pulse shape with pulse duration of 1 ms, an echo time (TE) of at least 2.45 ms, repetition time (TR) of 100 ms, nominal flip angle (*α*) of 15°, and 12 scans for signal averaging. The field of view (FOV) was 51.2 × 51.2 mm in the *x* and *z* direction, respectively, with a *k*-space consisting of 128 phase-encoded points in the *x* direction and 128 points in the readout direction along *z*. This resulted in a 2D image with slice thickness of 1 mm and an isotropic resolution of 400 μm, with a total experiment time of about 2.5 min.

### Data analysis

The phase map was generated from images with four different TEs (2.45, 2.50, 2.75, and 2.80 ms), and the UMPIRE^22^ algorithm was implemented in Python for phase-unwrapping to recover the field map.

### Susceptibility calculations

The susceptibility-induced modification to *B*_0_ caused by the paramagnetic lithium metal structure inside the voxel was calculated using a FFT method^23,24^ according to the equation

1 $${{h}}_{{\mathrm{obj}},{{z}}}\, = - {{H}}_0.{\mathrm{FT}}^{ - 1}\left[ {\frac{{{k}_{z}^2}}{{{k}^2}}.{\mathrm{FT}}({{\chi }})} \right]$$

Susceptibility values for the cells were obtained in two different ways: (1) Average susceptibility for the whole cell: the susceptibility value was obtained by performing a numerical fit to match the experimental magnetic field map with the predicted one from the cuboid. (2) In order to obtain the cathode susceptibility, the known susceptibility values for all other components were obtained from the literature^17^, and the volume fraction of the active cathode material was used to calculate the contribution from the cathode alone. The cell was weighed and measured to obtain the total mass and volume. Using the mean experimental susceptibility of the whole cell, the volume fraction of each component, and susceptibility values of all components except the cathode, one can calculate the susceptibility of the cathode changing with the oxidation state^14^. See Supplementary Table 1 and Supplementary Table 2 for the results.

### Average susceptibility for the whole cell

This is the method used for fitting the experimental field map to estimate the cell susceptibility:

A 3D model system, $\chi (x,y,z)$ was built to represent the susceptibility of the cell, with a cuboid representing the cell in the center of the simulation box. The simulation box was 256^3^ voxels, representing a volume of 102×102×102 mm with a 400-μm isotropic resolution to match the experimental conditions. A cuboid with dimensions 4.8×29.6×30.4 mm was used for the commercial cell and 2.4×35.2×51 mm for the RIT cell. The susceptibility values in the cell were set such that $\chi ({\mathrm{inside}}\,{\mathrm{battery}}) = \chi _{{\mathrm{cell}}}$ and $\chi = 0$ elsewhere.

The FFT susceptibility calculation method was used to predict the 3D magnetic field map around the cell in the model system, ${{B}}_{0,{\mathrm{sim}}}(x,y,z)$ (2D slide shown in Supplementary Fig. 2a).

A 2D slice of the simulated map was cropped (dotted box in Supplementary Fig. 2a) to match the dimensions of the experimental image. An additional mask was applied to select only regions that are non-zero in the experimental image, $B_{0,{\mathrm{exp}}}(x,y)$, which had been corrected by the reference image of the cell holder alone, i.e.,

$${B}_{0,{\mathrm{sim}}}^\prime (x,y) = \left\{ {\begin{array}{*{20}{c}} {B_{0,{\mathrm{sim}}}(x,y),B_{0,{\mathrm{exp}}}(x,y) \ne 0} \\ \hskip 3.9pc {0,B_{0,{\mathrm{exp}}}(x,y) = 0} \end{array}} \right.$$

The least-squares error between the simulated and experimental field maps was calculated, $|{{B}}_{0,{\mathrm{sim}}}^\prime (x,y) - B_{0,{\mathrm{exp}}}(x,y)|$, and summed, to provide a measure of the similarity between the two maps (Supplementary Fig. 2d).

The minimize function in the scipy package was used to fit the value of $\chi _{{\mathrm{cell}}}$ by repeating the calculation steps 1–4.

Supplementary Fig. 2d shows the difference between the experimental and simulation field maps for the optimally fitted value for $\chi _{{\mathrm{cell}}}$. The deviation was relatively large and illustrates the availability of further information beyond the basic model.

### Principal component analysis

PCA was performed on the magnetic field maps from the defected cells. The maps were first subtracted from the reference map that was obtained with only the holder, to yield the magnetic field map of the pouch cell alone. A mask was then applied to select only the central ~30×35-mm region of the maps, wherein the magnetic field varied relatively uniformly (the maps in Supplementary Fig. 3 illustrate this region) and where the magnetic field had been successfully recreated in each map. These masked maps were then flattened to 1D arrays and the data for all cells were assembled into a 2D array, on which PCA was subsequently performed using the Scikit-learn module in Python. To reproduce the loading plots in Supplementary Fig. 3, the PCA loadings were transformed back to the shape of the masked magnetic field maps.

### Data availability

The data that support the findings of this study are available from the corresponding author upon reasonable request.

## Electronic supplementary material

Supplementary Information
