# Supplementary material for: Rechargeable lithium-ion cell state of charge and defect detection by in-situ inside-out magnetic resonance imaging
Source: Nat Commun. 2018 May 3;9:1776. doi: 10.1038/s41467-018-04192-x (PMC5934497; doi:10.1038/s41467-018-04192-x)
Supplement: Supplementary file 1 — Supplementary Information [file 41467_2018_4192_MOESM1_ESM.pdf]

Supplementary Information for

Rechargeable Li-Ion Cell State of Charge and Defects by *In Situ* Inside-Out Magnetic Resonance Imaging

Ilott et al

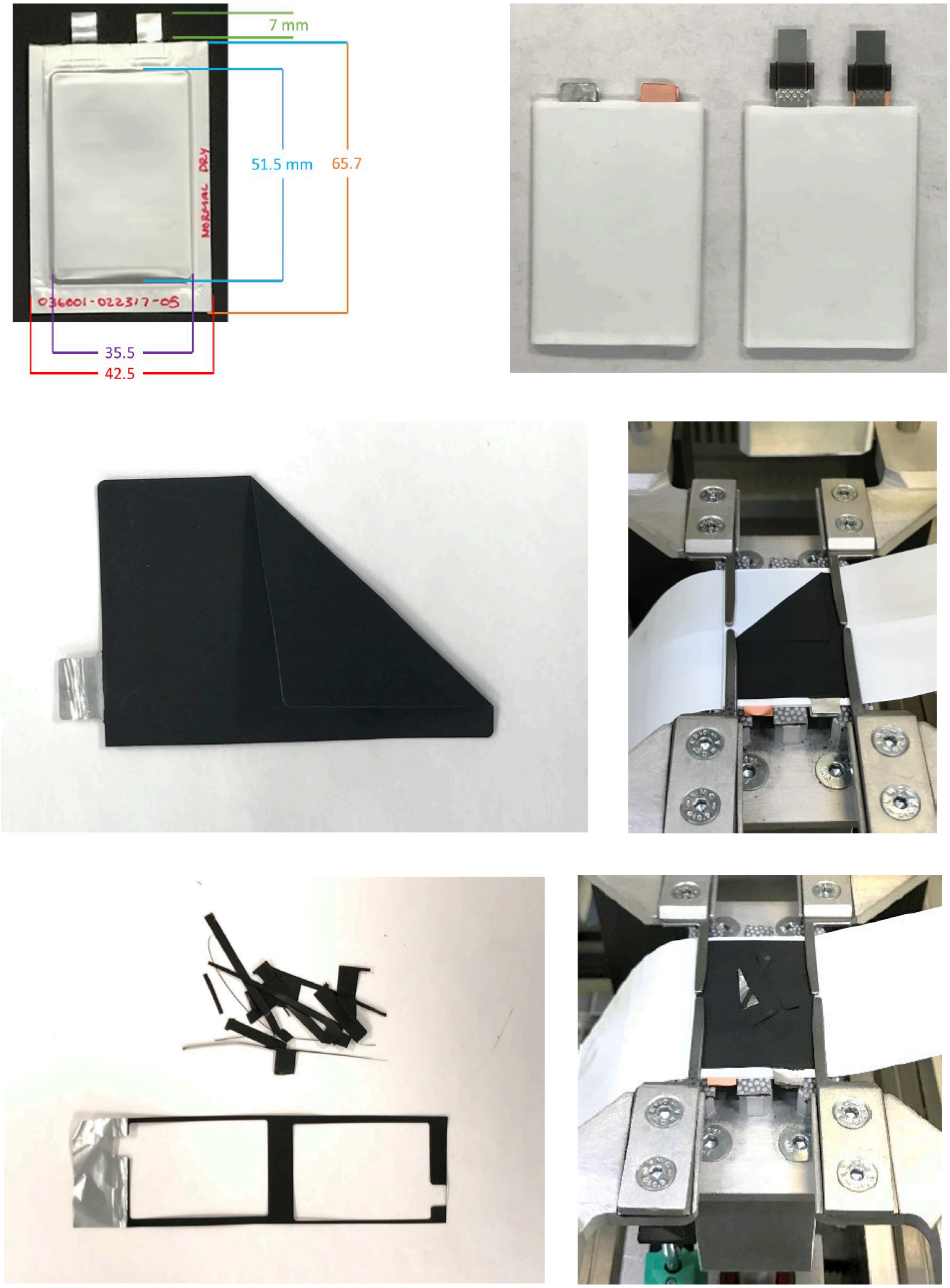

Supplementary Figure 1.
Preparation of defected RIT cells.

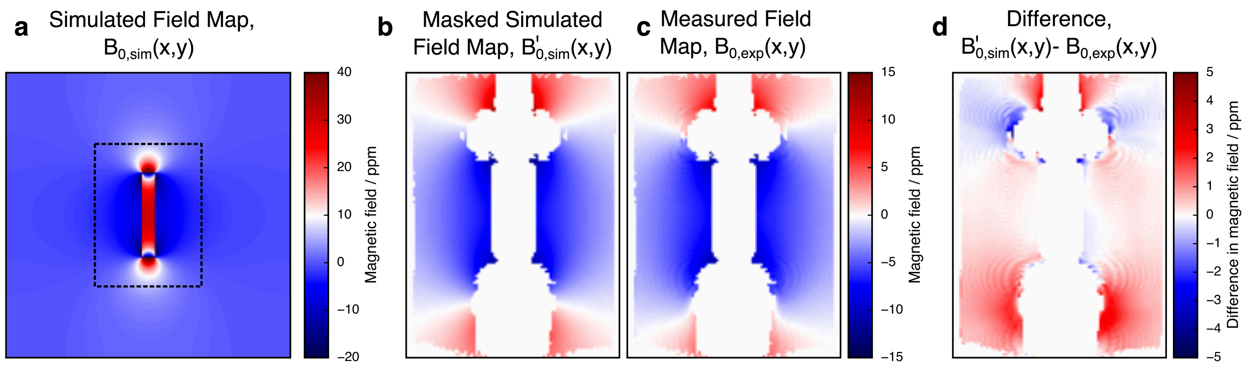

Supplementary Figure 2.

Illustration of susceptibility fitting process for the fully charged commercial cell.

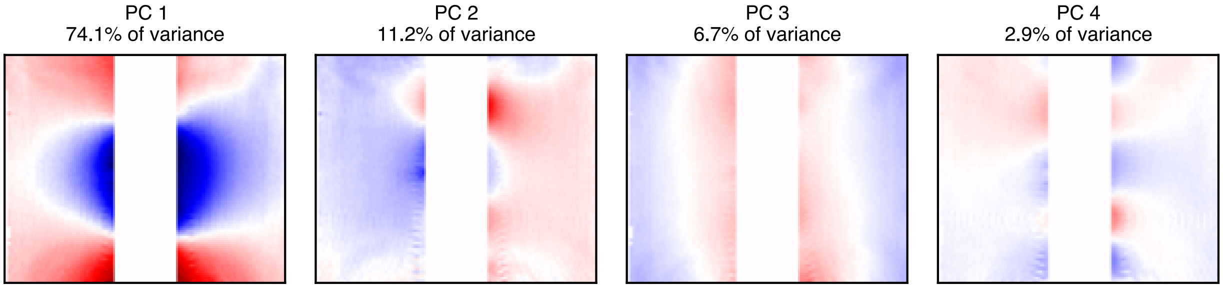

Supplementary Figure 3.

Loading plots for the first 4 principal components in the PCA.

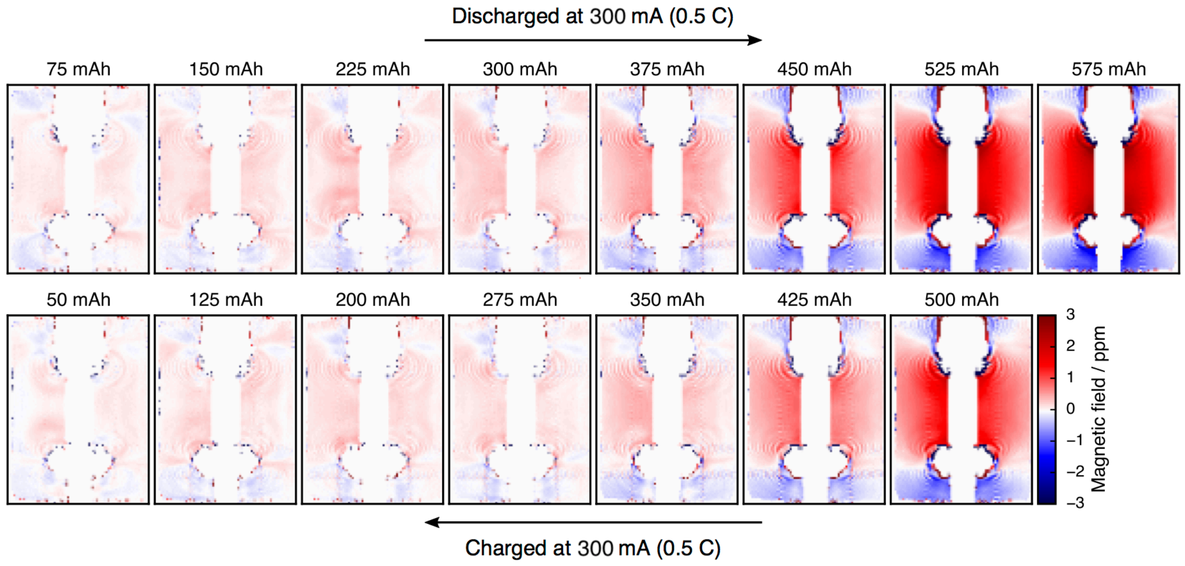

Supplementary Figure 4.

Series of magnetic field maps taken at intervals during discharge and then charge of the commercial cell. The plots are labeled by the discharge capacity of the cell at each step. The magnetic field is displayed with the fully charged cell as reference.

Supplementary Table 1.

Battery components and their composition (RIT cell). The volume fraction of each component is used to determine its contribution to the overall observed susceptibility. PVDF refers to polyvinylidene difluoride.

Cathode						
Composition (%wt)	$Li_{1.02}Ni_{0.50}Mn_{0.29}Co_{0.19}O_2$		Powder grade carbon black		PVDF	
	90		5		5	
Physical Properties	Volume (cm^3)	Density (gr cm^{-3})	Mass (gr)	# of layers	Thickness (cm)	Susceptibility χ_{volume}
	1.29	2.1	2.709	6	92×10^{-4}	Supplementary Table 2
**Anode**						
Composition (%wt)	Graphite		Powder grade carbon black		PVDF	
	92		2		6	
Physical Properties	Volume (cm^3)	Density (gr cm^{-3})	Mass (gr)	# of layers	Thickness (cm)	Susceptibility χ_{volume}
	1.65	1.2	1.82	13	42.5×10^{-4}	Supplementary Table 2
**Electrolyte**						
Composition (%wt)	1.2 M $LiPF_6$ EC:EMC 3:7					
	100					
Physical Properties	Volume (mL)	Density (gr cm^{-3})	Mass (gr)		Susceptibility χ_{volume}	
	0.95	1.56	1.48		1×10^{-6}	

Copper Current Collector				
Thickness (cm)	Volume (cm^3)	Density (gr cm^{-3})	Mass (gr)	Susceptibility χ_{volume}
1×10^{-3}	1.19×10^{-1}	8.92	1.06	-9.63×10^{-6}
**Aluminum Current Collector**				
Thickness (cm)	Volume (cm^3)	Density (gr cm^{-3})	Mass (gr)	Susceptibility χ_{volume}
1.20×10^{-3}	1.35×10^{-1}	2.7	3.65×10^{-1}	2.07×10^{-5}
**Separator**				
Thickness (cm)	Volume (cm^3)	Density (gr cm^{-3})	Mass (gr)	Susceptibility χ_{volume}
2.50×10^{-3}	4.10×10^{-1}	0.55	2.25×10^{-1}	1.9×10^{-6}

Supplementary Table 2.

Calculation of cathode susceptibility based on the state of charge: Using the physical measurements of the cell components (See Supplementary Table 1) and the anode susceptibility and the mean susceptibility of the cell at each state of charge, the cathode susceptibility was calculated.

Discharge capacity (mAh)	Anode susceptibility χ_{volume}	Mean susceptibility of the ell χ_{volume}	Cathode susceptibility χ_{volume}
0	-2.72×10^{-5}	1.08×10^{-4}	3.42×10^{-4}
95	-2.28×10^{-5}	1.22×10^{-4}	3.78×10^{-4}
125	-2.08×10^{-5}	1.31×10^{-4}	4.02×10^{-4}
185	-6.77×10^{-6}	1.52×10^{-4}	4.47×10^{-4}
215	5.56×10^{-6}	1.65×10^{-4}	4.70×10^{-4}
250	3.68×10^{-6}	1.66×10^{-4}	4.75×10^{-4}
